# Self-inflicted penetrating brain injuries with preserved neurological function: a case series

**DOI:** 10.1186/s41016-023-00328-1

**Published:** 2023-05-25

**Authors:** Shuja Shaukat, Syeda Mahrukh Fatima Zaidi, Adil Khatri, Mishal Shan Siddiqui, Muhammad Sameer Khulsai, Abdul Basit Ansari, Shabih Ayesha, Atiq Ahmed Khan, Muhammad Imran

**Affiliations:** 1 Department of Neurosurgery, Shaheed Mohtarma Benazir Bhutto Institute of Trauma, Karachi, Pakistan; 2 Department of Surgery, Dr. R K Pfau Civil Hospital, Karachi, Pakistan

**Keywords:** Penetrating brain injury, Self-harm, Head trauma

## Abstract

**Background:**

Penetrating trauma to the brain is a rare mode of self-harm in individuals with depressive psychosis. It may have variable presentations ranging from intact neurological status to non-survivable damage and the subjects may be surprisingly apathetic to pain. It is even unusual for such an injury to have an excellent prognosis despite coming late to clinical attention.

**Case presentations:**

We report two cases of patients with psychotic depression who attempted suicide by hammering nails into their heads. On imaging, deep penetration within the brain parenchyma was noted; however, neither case had any neurological deficit or symptoms attributable to brain trauma.

**Conclusions:**

Self-inflicted penetrating brain injuries with peculiar objects such as nails are rarely encountered in practice. They need prompt management for their removal and addressing the underlying mental health illnesses.

## Background

Penetrating brain injury is defined as a wound in which an object enters the cranium without making an exit [1]. Such incidents are largely reported being accidental or homicidal and quite unusual in routine neurosurgical practice. Self-inflicted injuries of this nature are often due to underlying mental health illnesses such as schizophrenia, acute psychosis, or major depressive disorder [2].

The burden of deliberate self-harm and suicide in South Asia, including Pakistan, remains largely unknown and under-reported [3]. Self-inflicted low-velocity penetrating trauma to the head is an extremely infrequent occurrence in our experience. Globally, only a handful of such cases have been described with presentations ranging from entirely asymptomatic individuals to life-threatening brain damage. The various tools reported include metallic rods, nails, screws, even intraocular chopsticks, and pencils [4–9]. However, repeated hammering of nails to the head is quite rare [10].

We report two such cases that we encountered in which the patients presented after driving nails into their heads while attempting suicide.

## Case series

### Case 1

A 26-year-old male was brought to the emergency department by his father when he incidentally noticed nails projecting from his son’s head. The patient had hammered four nails into his head, each measuring 10 cm, with a brick under the influence of alcohol two days before presentation. He continued to function normally while covering them with a cap at home. He also had a prolonged history of untreated depression and alcohol abuse with three attempted suicides in the past.

**Fig. 1:**
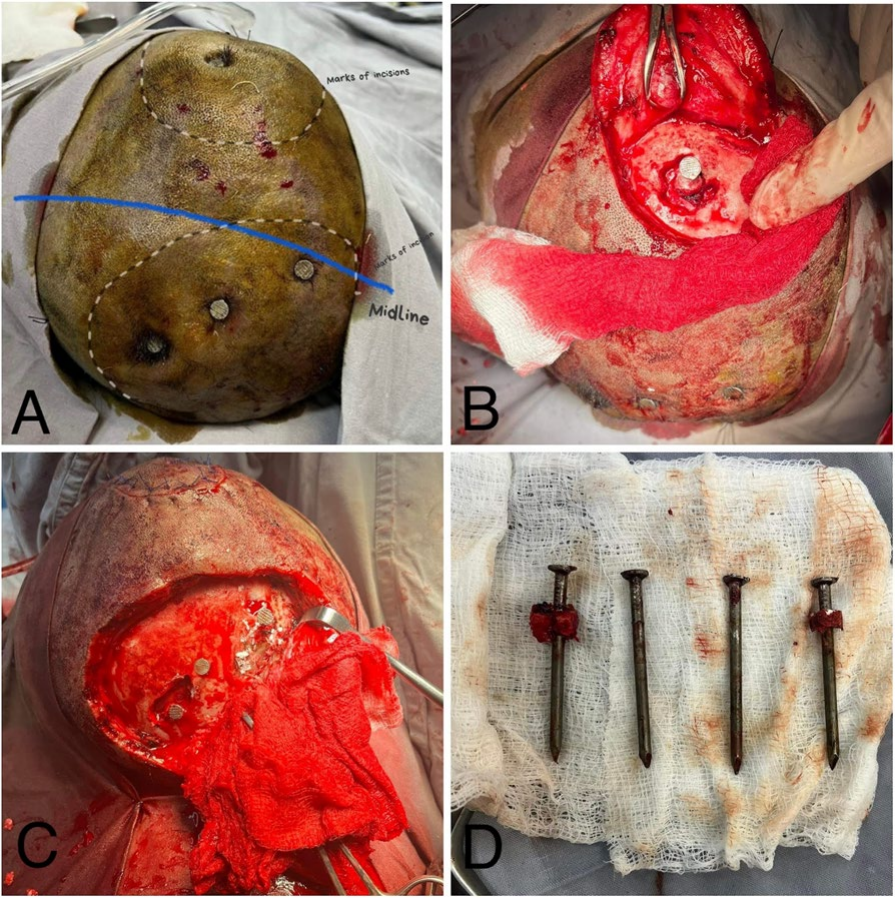
Case 1. **A** Four nails inserted under his scalp with multiple puncture wounds with marked midline and area of incisions. **B** C-shape incision given left posterior most nail. **C** C-shaped incision given around frontoparietal nails. **D** Four 10 cm long nails extracted from the patient

**Fig. 2:**
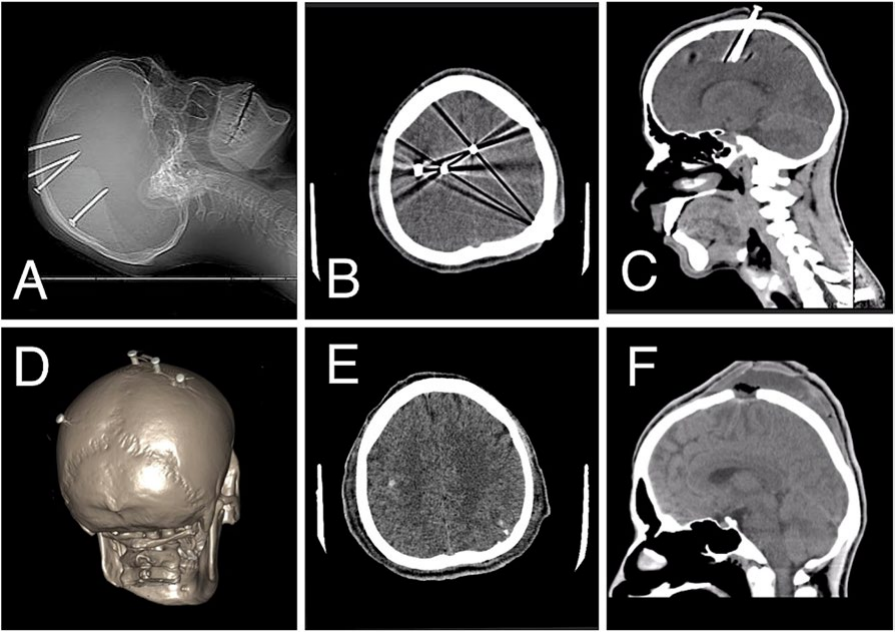
**A**, **B** Pre-operated CT brain plain axial view showing penetrating objects. **C** Pre-operative sagittal CT brain plain showing ¼ foreign penetrating object not associated with underlying hemorrhage. **D** Three-dimensional Computed tomography skull showing all 4 penetrating objects, 1 penetrating left parietal bone, and the remaining 3 penetrating right parietal bone. **E** Post-operative axial CT brain plain, where Post-operative pinpoint hemorrhage can be seen. **F** Post-operative CT brain plain sagittal view showing craniectomy done around penetrating object and subgaleal collection. No Postoperative intraparenchymal hemorrhage was seen

On arrival, he was vitally stable and without any gross neurological deficits. However, he was completely apathetic toward pain. Inspection of the scalp revealed 3 nails over the right mid-parietal region and 1 nail at the left parieto-occipital region. There were several other healed puncture wounds of which the patient had no memory (see Fig. 1). Marks of self-harm on the neck and wrist were also present. A non-contrast CT scan was performed which showed 3 metal artifacts penetrating the right frontoparietal region, 1 crossed midline and the tip of another was around the occipital horn of the lateral ventricle (see Fig. 2).

Emergency removal of the nails was carried out the same day. A C-shaped incision was given at the right frontoparietal region and the flap was carefully raised around the three right-sided nails. The nails, thus loosened, were then retrieved. A similar craniectomy was performed through a curvilinear incision posteriorly on the left side, and the left nail was removed (see Fig. 1). There were no peri- or postoperative complications. The patient had an uneventful recovery, and he was managed with prophylactic antibiotics and anti-epileptic drugs. Upon psychiatric consultation, he was diagnosed with acute psychotic depression and, thus, shifted to the psychiatry ward where he was started on oral ecitalopram 20 mg and alprazolam 0.5 mg. The patient received a 6-month course of antidepressants and continued anti-epileptic medication with no post-operative complications or neurological deficits, and was scheduled for a follow-up after 6 months.

### Case 2

A 24-year-old incarcerated male was brought to the emergency room by the police four hours after he had hammered a 10-cm long nail in the middle of his head by repeatedly striking his head against the cell’s wall. He had a history of a similar attempt 1 month back, which resulted in a deep scalp laceration.

**Fig. 3:**
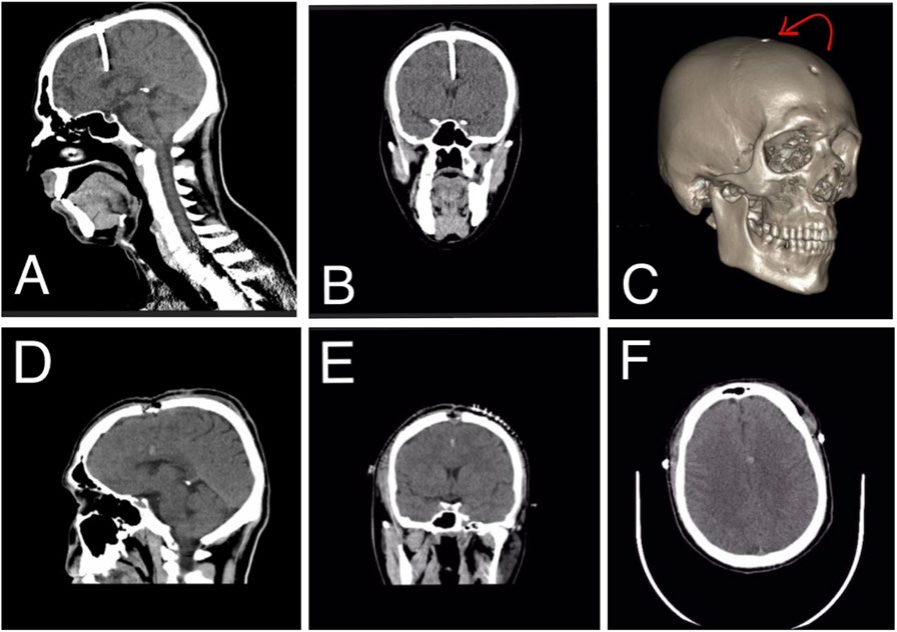
**A** Preoperative sagittal view of CT brain plain showing single nail projecting at the level of the junction between anterior 1/3rd and middle 1/3rd of superior sagittal sinus, no associated hemorrhage or signs of raised ICP. **B** Preoperative coronal view of CT brain plain showing midline penetrating object just above frontal horn of lateral ventricle. **C** Computed tomography three-dimensional reconstruction of skull showing foreign body entry (red arrow). **D** Postoperative CT brain plain sagittal view showing craniectomy, and small hemorrhagic point, while no remnants of the foreign body were seen. **E** Postoperative coronal CT brain plain. **F** Post-operative axial CT brain plain

On examination, he was fully conscious with intact neurological function. A nail was impacted perpendicularly over the frontal region in the midline. There were no derangements in his laboratory parameters. A non-contrast CT scan revealed a hyperdense linear object at the vertex involving the frontal bone in the midline and extending deep into the superior sagittal sinus and brain parenchyma. On the brain window, an interhemispheric bleed and a left frontal contusion were also appreciated (refer to Fig. 3).

Emergency craniectomy and nail removal was subsequently attempted. Bi-coronal flaps were raised, craniectomy was done around the nail, and the foreign body was extracted. Postoperative recovery was unremarkable. The psychiatry team was taken on board and a diagnosis of acute psychotic depression was made. The patient was discharged back to central jail on the 6th postoperative day without any postoperative complications After evaluation, the patient was commenced on anti-depressant and anti-epileptic therapy and scheduled for a follow-up after two weeks but was subsequently lost to follow-up.

## Discussion

Penetrating brain injury is very rarely self-imposed and may carry a grave prognosis. Various tools for self-harm have been described in the literature and they range from the commonly used knives to the most bizarre objects such as pencils, chopsticks, nails, metal rods, and screwdrivers [4–9]. In our cases, metallic nails were inserted with the aid of a brick in the first case and a wall in the other. The clinical presentation of penetrating brain injuries vary depending on the nature and site of injury, the velocity of the penetrating object, the angle of penetration, and the overlying calvarial thickness [11]. Unlike firearm injuries and high-speed motor vehicle accidents, low-velocity penetrations cause a more localized damage in the absence of any heat dissipation or shearing forces. The neurological damage that results is largely predictable based on the region that is injured. It may include altered consciousness, seizures, or focal neurological deficits. Alternatively, the patients may be minimally or entirely asymptomatic [11, 12]. In both cases, there was no neurological dysfunction despite the nails being inserted deep within the parenchyma as the eloquent areas of the brain were spared.

An underlying psychiatric condition has been a frequent accompaniment of such cases of deliberate self-harm. Schizophrenia, bipolar disorder, major depressive disorder, and acute drug-induced psychosis have all been implicated. In one case, a young female attempted suicide by shoving a metal rod in her head after experiencing auditory hallucinations [6]. Another report described a female who stabbed herself with a knife to get rid of her chronic headache [5]. In our cases, a formal psychiatric evaluation revealed underlying psychotic depression in both patients; both had a history of previous suicidal attempts and longstanding, untreated depression.

It is very atypical to encounter repeated self-imposed trauma to the head with the same tool, as occurred in our first case. A similar event was narrated by James et al., in which a 44-year-old male had driven 10 nails of various sizes in his head over a span of 3 months. His neurological functions were preserved until the last nail which led to left-sided weakness and gait unsteadiness [10]. In our case, the patient also presented late, i.e., 2 days, after the attempt. He continued to cover his head with a cap and continued with his routine activities. It was only by accident that the nail came to his family’s attention when his cap was removed.

Another case by Salar et al. reported a 36-year-old male who was found semi-conscious with 11 nails protruding from his head. While they were successfully removed without any focal neurological deficits, follow-up at 1 year showed impairment of memory, and a diagnosis of frontal lobe syndrome was made [13]. Although a thorough assessment of higher executive functions was not conclusive in our patients owing to their co-existent psychosis, such cases should be followed up for any late complications including epilepsy, pseudoaneurysm formation, and residual deficits. The insertion of metallic foreign bodies also risks tetanus and bacterial meningitis, both of which need prompt prophylaxis and were initiated in our patients. In a case series by Ohaegbulam et al., meningitis was the cause of death after the nail was successfully extracted from the patient’s brain [14].

Depending on the depth of penetration and nearby structures, the foreign bodies can be extracted either under local anesthesia or via craniectomy under general anesthesia. We opted for the latter given the deep-seated location of the nails in both of our patients. In cases where multiple such foreign bodies are found projecting in various directions, it may be wiser to perform separate mini craniotomies instead of a single craniotomy to manipulate the nails. This strategy may minimize the damage caused by nail mobilization and manipulation [2].

## Conclusion

Self-inflicted nail injury to the head is rare and may be encountered in patients with depressive psychosis. Despite the gruesome presentation, they may be surprisingly asymptomatic and pain-free. However, they require urgent neurosurgical intervention and prompt psychiatric help, for optimum outcomes, and prevention of future occurrences.

## Data Availability

Not applicable.

## References

[1] Modi M, Arivazhagan A, Bharath RD, Rao MB, T M, AM M (2014). Penetrating brain injury with Machete, stuck to calvarium: Hurdles in imaging and solutions. J Neurosci Rural Pract.

[2] Arici L, Akgun B, Kaplan M, Yilmaz I (2012). Penetrating head trauma with four nails: An extremely rare case. Ulus Travma Acil Cerrahi Derg.

[3] Shekhani SS, Perveen S, Hashmi D-S, Akbar K, Bachani S, Khan MM (2018). Suicide and deliberate self-harm in Pakistan: a scoping review - BMC psychiatry. BMC Psychiatry.

[4] Kishore K, Agarwal A, Kumar A, Dahiya S, Bharti P, Sahu S (2010). Management of unusual case of self-inflicted penetrating craniocerebral injury by a nail. J Emerg Trauma Shock.

[5] Qazi Z, Ojha B, Chandra A, Singh S, Srivastava C, Verma N (2017). Self inflicted stab with a knife: an unusual mode of penetrating brain injury. Asian J Neurosurg.

[6] Panigrahi S, Mishra SS, Das S, Pattajoshi AS (2013). Self-inflicted penetrating brain injury by an iron rod in a psychiatric patient: Case report and literature review. Indian J Neurotrauma.

[7] Lee Y, Ma K, Lai H, Pan N, Wong T, Kwan H (2017). Self-inflicted transorbital brain injury by chopsticks in a patient with acute psychosis. Xianggang Yi Xue Za Zhi.

[8] Cvetković D, Živković V, Damjanjuk I, Nikolić S (2018). The pen is mightier than the sword”—suicidal trans-orbital intracranial penetrating injury from a pencilcite. Forensic Sci Med Pathol.

[9] Cemil B, Tun K, Yigenoğlu O, Kaptanoğlu E (2009). Attempted suicide with screw penetration into the cranium. Ulus Travma Acil Cerrahi Derg.

[10] James G, Blakeley CJ, Hashemi K, Channing K, Duff M (2006). A case of self-inflicted craniocerebral penetrating injury. Emerg Med J.

[11] Young L, Rule GT, Bocchieri RT, Walilko TJ, Burns JM, Ling G (2015). When physics meets biology: Low and high-velocity penetration, blunt impact, and blast injuries to the brain. Front Neurol.

[12] Makoshi Z, AlKherayf F, Silva VD, Lesiuk H (2016). Nail gun injuries to the head with minimal neurological consequences: a case series - journal of medical case reports. J Med Case Rep.

[13] Salar G, Costella GB, Mottaran R, Mattana M, Gazzola L, Munari M (2004). Multiple craniocerebral injuries from penetrating nails: case illustration. J Neurosurg.

[14] Ohaegbulam SC, Ojukwu JO (1980). Unusual craniocerebral injuries from nailing. Surg Neurol.

